# Protective efficacy of a ‘pan-fungal’ vaccination strategy against experimental *Pneumocystis* infection in drug-immunosuppressed macaques

**DOI:** 10.3389/fimmu.2025.1729080

**Published:** 2026-01-16

**Authors:** Whitney Rabacal, Anna Hu, Gabrielle Kirton, Taylor I. Chapman, Daniel Wychrij, Kwadwo O. Oworae, Karen A. Norris

**Affiliations:** Center for Vaccines and Immunology, Department of Infectious Diseases, University of Georgia, Athens, GA, United States

**Keywords:** dexamethasone, immunosuppression, macaque, non-human primate, NXT-2, pan-fungal vaccine, pneumocystis

## Abstract

**Introduction:**

**Pneumocystis* jirovecii* causes life-threatening fungal pneumonia (PJP) and other serious pulmonary sequelae in HIV infected individuals and other immunocompromised populations. In recent years, while the frequency of PJP has declined in HIV infected individuals treated with anti-retroviral therapies, the incidence has increased among non-HIV populations due to the expanding use of corticosteroids and other immunomodulatory agents to treat immune-mediated inflammatory diseases and hematologic and solid malignancies. Despite the success of trimethoprim-sulfamethoxazole (TMP-SMX) prophylaxis, patients who are unable to tolerate treatment, take drugs where TMP-SMX is contraindicated, or experience breaks in daily compliance remain at risk. Immunocompromised populations would benefit from vaccine strategies that reduce morbidity and mortality due to acute PJP.

**Methods:**

Herein, we used a newly established non-human primate (NHP) model of *Pneumocystis* infection in the context of drug-induced immunosuppression to test the immunogenicity and protective efficacy of a vaccine strategy administered prior to and throughout drug-induced immunosuppression using the ‘pan-fungal’ vaccine candidate NXT-2a. Longitudinal blood and bronchoalveolar lavage sampling was performed to monitor anti-NXT-2a antibody titers, lymphocyte populations, and infection status.

**Results:**

Immunization with NXT-2a prior to immunosuppression induced robust humoral immune responses in healthy outbred macaques. Subsequent therapeutic boosting throughout drug-induced immunosuppression prevented protective antibody titer decline. Our collective vaccination strategy provided significant protection against *Pneumocystis* infection throughout the duration of the study.

**Discussion:**

These studies demonstrate the efficacy and feasibility of an NXT-2a based vaccination strategy in a NHP model with a planned immunosuppressive regimen. This strategy may be further applied toward other opportunistic fungal pathogens, such as *Candida* spp. and *Aspergillus* spp. in similarly immunosuppressed populations.

## 1 Introduction

*Pneumocystis jirovecii* is an opportunistic fungal pathogen that can cause life-threatening pneumonia (PJP) and has been associated with a number of chronic lung diseases including chronic obstructive pulmonary disease (1), severe asthma (2), cystic fibrosis (3), and interstitial lung disease (4). Transmission is airborne and occurs following exposure to other *Pneumocystis* carrying individuals (5). Asymptomatic colonization and clearance are common among immunocompetent populations, but in immunosuppressed individuals, persistent colonization can lead to pneumonia. In the pre-anti-retroviral era of the HIV epidemic, PJP was a leading cause of morbidity and mortality in individuals with HIV. In recent years, the incidence of PJP has declined among people with HIV but has steadily increased among non-HIV immunosuppressed individuals (6, 7). Populations at high risk include those receiving corticosteroids and other immunomodulatory therapies for immune-mediated inflammatory diseases, organ transplantation, and cancer (8, 9). PJP in persons without HIV is generally associated with a lower organism burden and a more acute and fulminant disease course than in persons with HIV (10–12), complicating efficient diagnosis and successful treatment. Even with clinical intervention, mortality associated with PJP remains high (25%) and worsens if ICU admission is required (58%) (13). Trimethoprim-sulfamethoxazole (TMP-SMX) is an effective treatment and prophylactic agent against *Pneumocystis* pneumonia in immunocompromised individuals; however, efficacy is often limited due to drug-drug interactions, treatment-limiting adverse events, and breakthrough infections (14–16). Notably, treatment with TMP-SMX does not prevent re-infection and widespread use has raised concerns over emerging drug resistance (15, 17). Therefore, strategies that can promote anti-*Pneumocystis* immunity, and are well-tolerated alongside pharmaceutical regimens are necessary to reduce the high rates of morbidity and mortality associated with PJP.

To address this need, our laboratory has previously developed a ‘pan-fungal’ vaccine candidate, NXT-2. NXT-2 is a 90-amino acid consensus peptide, based on a conserved region of the kexin-like protein KEX1 from the pathogenic fungi *Pneumocystis jirovecii*, *Aspergillus fumigatus*, *Candida albicans*, and *Cryptococcus neoformans *(18, 19). The development of NXT-2 was based on extensive prior research demonstrating the importance of antibodies against *Pneumocystis* KEX1 (PC.KEX1) in the control of *Pneumocystis* associated disease. Due to natural exposure, most individuals are PC.KEX1-serpositive, and high antibody titers against this antigen correlate with a decreased frequency of *Pneumocystis* associated disease among HIV infected individuals (20) and in a non-human primate (NHP) model of *Pneumocystis* and HIV co-infection (21). Kling et al. further demonstrated that immunization with recombinant PC.KEX1 boosts humoral memory in immunocompetent macaques and is protective against subsequent *Pneumocystis* infection during simian immunodeficiency virus (SIV)- immunosuppression (22). Interestingly, proof-of-concept studies demonstrate that therapeutic immunization with PC.KEX1 during SIV and methylprednisolone/tacrolimus -induced immunosuppression boosts antibody recall responses (23, 24) and can help to maintain immunity in NHP model of *Pneumocystis* and SIV co-infection (24). Like PC.KEX1, immunization with NXT-2 is highly immunogenic in both mice and NHPs, and induces protective immunity against a range of experimental fungal infections, including systemic candidiasis and pulmonary aspergillosis in immunosuppressed murine models, murine vulvovaginal candidiasis, and in a NHP model of *Pneumocystis* and SIV co-infection (18, 25).

We recently developed a novel NHP model to study the efficacy of NXT-2-based biologics in the context of severe drug-induced immunosuppression and natural airborne exposure to *Pneumocystis *(26). Herein, we evaluated the immunogenicity of NXT-2 in healthy macaques and evaluated its protective efficacy against *Pneumocystis* infection during drug-induced immunosuppression with therapeutic boosting. These studies present a strategy for immunizing individuals who are at risk of developing *Pneumocystis* pneumonia following immunosuppressive therapies.

## 2 Materials and methods

### 2.1 Animals

Eight Japanese macaques (*Macaca fuscata*) aged 4–9 years were obtained from Oregon National Primate Research Center (ONPRC) and randomly assigned to vaccinated (n=4, 3 females, 1 male) or sham control (n=4, 2 females, 2 males) cohorts. All studies were approved by the Institutional Animal Care and Use Committee (IACUC) of the University of Georgia. All animal studies were performed in the University Research Animal Resources Facility, at the University of Georgia, an American Association for the Accreditation of Laboratory Animal Care (AAALAC) accredited facility. The care and use of laboratory animals at the University of Georgia are in accordance with the principles and standards set forth in the Principles for Use of Animals (NIH Guide for Grants and Contracts), the Guide for the Care and Use of Laboratory Animals, the provision of the Animal Welfare Acts (P.L. 89–544 and its amendments). Compliance is validated by the UGA IACUC and regular inspections by USDA inspecting veterinarians. Prior to admission to the study, all animals underwent physical examination and were screened and found negative for simian retroviruses (SIV, SRV, and STLV).

### 2.2 Recombinant expression and purification of NXT-2a antigen

The design and expression of NXT-2 was previously reported (18). To generate an affinity tag-free NXT-2 construct, the 90-amino acid pan-fungal consensus sequence first described in Rayens et al. (18) followed by two stop codons (ochre and opal) were cloned into the pET-28b(+) vector (Novagen) using the restriction sites NcoI and BamHI by GenScript. This vector was then used to transform chemically competent *Escherichia coli* BL21(DE3) pLyS cells (Thermo Fisher) according to the manufacturer’s instructions. This resulted in an affinity tag-free recombinant protein, NXT-2a, expressed as 5’- MGPDDGKTMEGPDILVLRAFINGVQNGRDGKGSIYVFASGNGGGFEDNCNFDGYTNSIYSITVGAIDRKGLHPSYSEACSAQLVVTYSSGSG-3’. Following subculture in BBL Select APS LB Broth base (BD Biosciences) with kanamycin (40µg/ml) and chloramphenicol (34µg/ml), protein expression was induced for four hours at 37°C in a final concentration of 0.5mM IPTG where the protein was found to be expressed primarily in inclusion bodies. Inclusion bodies were isolated by lysing cell pellets with CelLytic B (Sigma) and washing the pellets three times with diluted CelLytic B (1:10 dilution with water) according to the manufacturer’s instructions. Inclusion body pellets were resuspended in buffer A (6M Urea, 20mM Tris-HCl, pH 8.0) and nutated for two hours at room temperature before storing the suspension overnight at 4°C. The next day, the suspension was centrifuged at 10,000g for 15 minutes at 4°C. The supernatant was then collected and filtered over a 0.2µm filter. Supernatants were then run over a HiTrap Capto Q column and NXT-2a was eluted through a gradient of buffer B (1M NaCl 6M urea 20mM Tris-HCl, pH 8.0) using the AKTA Pure FPLC system (Cytiva). Following this initial anion exchange chromatography capture step, NXT-2a enriched fractions were then pooled and concentrated by diafiltration using a 3 kDa MWCO filter (Merck Millipore). Size exclusion chromatography was performed as a final polishing step. Pooled anion exchange fractions were run over a Superdex 75 Increase 10/300 column (Cytiva) and eluted in 6M Urea, 250mM NaCl, 20mM Tris-HCl, pH 8.0 buffer. Protein refolding and buffer exchange were performed by dialyzing the final pooled fractions overnight in 1x PBS using the Pur-A-Lyzer Maxi Dialysis Kit (Sigma-Aldrich) with two changes of buffer. The final protein containing fractions were then analyzed for purity by Coomassie and Western blotting as previously described (18). SDS-PAGE was performed by running 5µg fractions on a 4% stacking/15% resolving polyacrylamide gel. Proteins were then transferred to a 0.2µm nitrocellulose membrane, blocked in 5% nonfat milk in PBS-T (0.05% Tween-20), and incubated with anti-NXT-2 hyperimmune NHP plasma (1:10,000). Detection was performed using goat anti-monkey IgG HRP (ThermoFisher, 1:10,000), SuperSignal PicoWest Plus chemiluminescence substrate (ThermoFisher), and a ChemiDoc (BioRad) imaging system. NXT-2a was used for immunizations and enzyme-linked immunosorbent assays (ELISA). The final purified pool of NXT-2a was endotoxin-tested using the Pierce Chromogenic Endotoxin Kit (ThermoFisher) prior to *in vivo* use.

### 2.3 Preparation of NXT-2a vaccine, immunization of macaques, and drug-induced immunosuppression

Seven macaques were intramuscularly immunized with prepared NXT-2a vaccine (n=4) or sham vaccine (n=3) at baseline and boosted 6 weeks later. Each macaque received 500µl prepared vaccine comprised of NXT-2a (100µg) + Alhydrogel 2% (InvivoGen, 0.5mg Al^3+^) diluted in sterile PBS or Alhydrogel 2% (0.5mg Al^3+^) alone in PBS that was rocked overnight at 4°C to encourage antigen and matrix binding. Two weeks after boosting, animals were treated daily with dexamethasone (West-Ward Pharmaceuticals, 1.3-1.6mg/kg/day) until the end of the study to induce immunosuppression. A fourth sham control animal, 39635, received no vaccines prior to and following the start of dexamethasone treatment.

### 2.4 Pneumocystis challenge

*Pneumocystis* cannot be reliably cultured *in vitro*. Immunocompetent individuals and macaques may be transiently or asymptomatically colonized but are only susceptible to *Pneumocystis* infection when immunosuppressed (26–28). We have previously reported that immunosuppressed macaques may be infected by natural airborne transmission of *Pneumocystis* from other *Pneumocystis* positive “seeder” (infected or colonized) macaques by co-housing (22, 24, 26). The “seeder” animal, sham control 39635, used in this experiment was a non-vaccinated macaque that was treated with dexamethasone 4 weeks prior to immunosuppression of the remainder of the cohort. 39635 became persistently colonized by 4 weeks of immunosuppression, coinciding with the start of dexamethasone treatment in the remainder of the cohort.

### 2.5 Sample collection

Blood and BAL samples were collected and processed as previously described (21, 27). Blood was collected at baseline and then every two weeks until the end of study to monitor anti-NXT-2a IgG antibody levels and lymphocyte kinetics. BAL procedures were performed with 20ml of sterile PBS. BAL samples were collected every 4 weeks for the first 8 weeks of dexamethasone treatment and then every 2 weeks until the end of the study to monitor *Pneumocystis* infection status and lymphocyte kinetics. In some cases where monitoring results were found to be inconclusive, BAL collection was repeated the following week.

### 2.6 Anti-NXT-2a ELISA

ELISA assays were performed using NXT-2a coated plates as previously described (22, 23). Microtiter plates (Immulon 4HBX; Thermo Fisher Scientific) were coated with recombinant NXT-2a (50µl/well at 5µg/ml in 1x PBS) over night at 4°C. After washing twice with PBS-T plates were blocked with blocking buffer (5% non-fat dry milk in PBS) for 1 hour at room temperature. Plates were then washed twice with PBS-T, dried, and then stored at -20°C for up to 6 months prior to use. To measure anti-NXT-2a antibody titers, heat-inactivated plasma samples were initially diluted 1:100 in blocking buffer and two-fold serial dilutions were made prior to adding 50µl of diluted sample in NXT-2a coated plates. Plates were then incubated overnight at 4°C. The next day, plates were washed four times with PBS-T and incubated with 100µl/well of goat anti-monkey IgG-HRP secondary antibody (Nordic Immunology) diluted 1:10,000 in blocking buffer for 1 hour at 37°C. Plates were then washed six times with PBS-T and visualized with 100µl/well TMB (BD Biosciences), the reaction stopped with 50µl/well of 1M H_2_SO_4_ and read at 450nm. Normal (uninfected, *Pneumocystis*-negative determined by antibody titer) macaque plasma was used as a negative control, and archival samples from a vaccinated animal with a known titer were used as a positive control as internal controls on all assay plates.

### 2.7 Evaluation of *Pneumocystis* infection

BAL samples were processed, and DNA was extracted from BAL pellets as previously described (26). All BAL processing, DNA extraction and PCR steps were performed under sterile conditions in either a biosafety cabinet or a PCR workstation (Fisher Scientific) to prevent ambient *Pneumocystis* contamination. *Pneumocystis* infection was defined as detection of the *Pneumocystis* mitochondrial large subunit rRNA gene (mtLSU) by PCR (26, 27). *Pneumocystis* colonization was defined as detection of *Pneumocystis* DNA by nested PCR of the 1st round PCR product only (2nd round PCR positive, +) (21, 26). A β-globin PCR was performed as a control for DNA quality. mtLSU and β-globin PCR reactions were performed using 1µg of BAL template DNA. PCR products were run on a 1.5% agarose gel with SYBR Safe gel stain (ThermoFisher) and visualized with the ChemiDoc (BioRad) imaging system. Semi-quantitative densitometry analysis was performed using Image J (NIH) to compare the ratio of mtLSU: β-globin between longitudinal timepoints. *Pneumocystis* infected animals with distinctly positive mtLSU gel products displayed a ratio of mtLSU: β-globin greater than 0.25 when analyzed by densitometry.

### 2.8 Flow cytometry

Blood and BAL samples were collected and processed as previously described (26, 27). Red blood cells were lysed by treating whole blood with red blood cell lysis buffer (150mM NH_4_Cl, 10mM NaHO_3_, 115µM EDTA). Cells were stained in FACS buffer containing 20% FBS, 2% human sera, 2% goat serum, 5mM EDTA, and 0.05% sodium azide in 1x PBS. FITC anti-CD3 (SP34), PE anti-CD8 (RPA-T8) antibodies were purchased from BD Biosciences. APC anti-CD4 (OKT4), and APC-Cy7 anti-CD20 (2H7) were purchased from BD Biolegend (San Diego, CA). After antibody staining, cells were lysed and fixed in BD FACS Lysing Solution (BD Biosciences) to eliminate residual red blood cells and then stored in 1% paraformaldehyde until sample acquisition. Standard flow cytometric procedures were used to acquire data on a NovoCyte Quanteon flow cytometer (Agilent Technologies, Santa Clara, CA). Analysis was performed using FlowJo analysis software (BD Biosciences). The gating strategy used was as previously described in Rabacal et al. (26).

### 2.9 Statistical analysis

All statistical analyses were performed using GraphPad Prism (GraphPad Software, La Jolla, CA). Longitudinal changes in lymphocyte populations in all dexamethasone treated animals (NXT-2a immunized and sham controls combined) over time were analyzed using repeated measures mixed modeling and Dunnett’s test for multiple comparisons to identify values that differ significantly from baseline values. Timepoint specific differences in lymphocyte populations between NXT-2a immunized and sham control cohorts were analyzed by multiple Mann-Whitney tests. Log-rank test was used to analyze *Pneumocystis* infection incidence curves.

## 3 Results

### 3.1 Humoral responses in healthy Japanese macaques and following therapeutic boosting during dexamethasone induced immunosuppression

**Figure 1:**
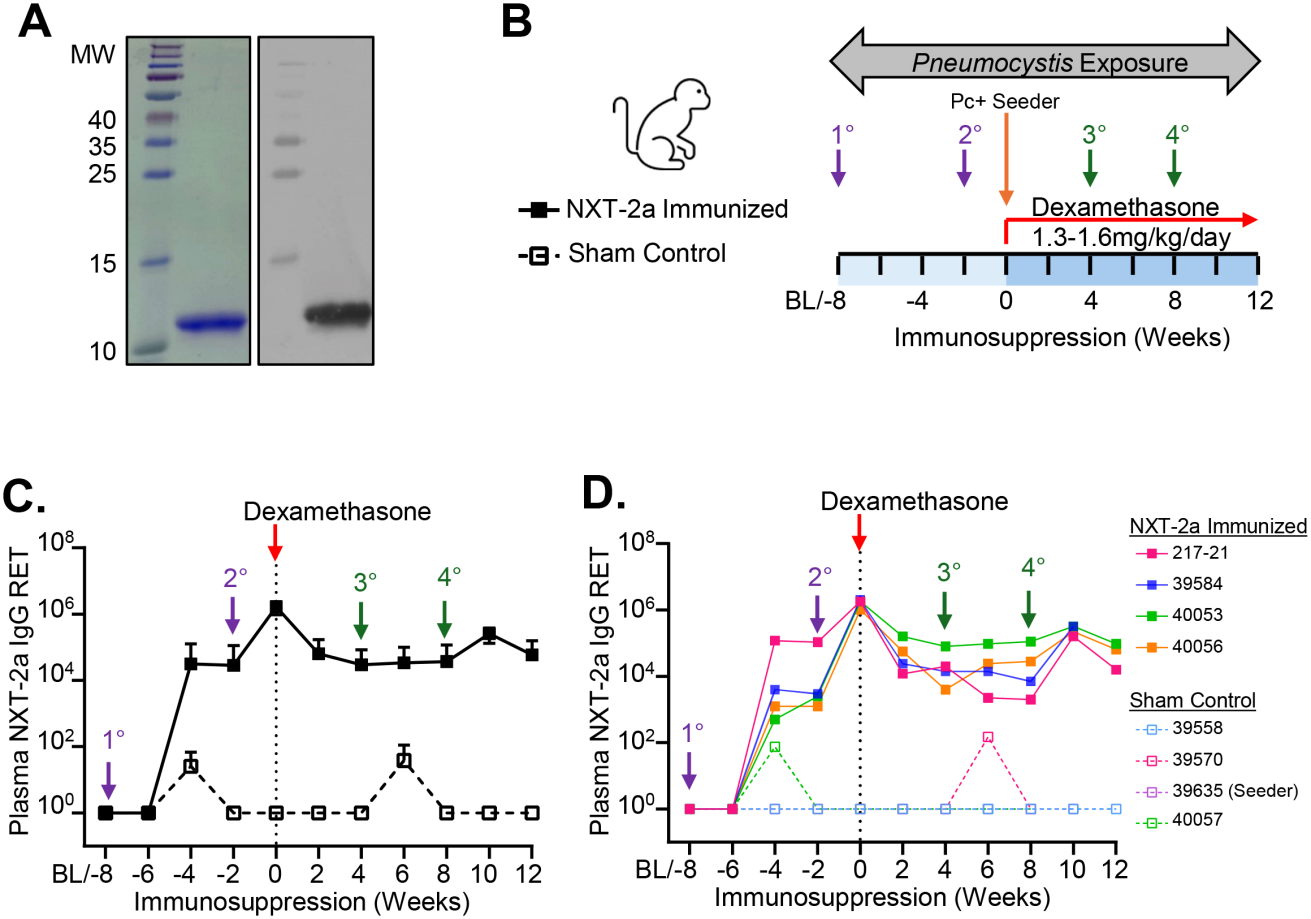
Humoral responses in NXT-2a immunized macaques prior to and following dexamethasone induced immunosuppression. **(A)** Coomassie stained gel (left) and Western blot of recombinant NXT-2a (~10kDa) used for immunization and ELISA assays. Immunoblotting was performed using polyclonal sera from a macaque immunized with NXT-2 (affinity tagged construct). **(B)** Study design of immunization, immunosuppression, and *Pneumocystis* infection in Japanese macaques. Purple arrows indicate the primary vaccine series administered prior to immunosuppression. Green arrows indicate the secondary vaccine series administered and therapeutically throughout immunosuppression. A non-vaccinated animal, sham control 39635 initiated dexamethasone treatment ~4 weeks prior to the remainder of the cohort. 39635 became *Pneumocystis* colonized (Pc+) by 4 weeks of immunosuppression when the remainder of the cohort initiated dexamethasone treatment and functioned as a “seeder” to facilitate the spread of *Pneumocystis* to other co-housed animals (orange arrow). Cross transmission of *Pneumocystis* among co-housed macaques is indicated by the double arrow. **(C)** Kinetics of NXT-2a specific reciprocal endpoint titers (RET) in the plasma of NXT-2a immunized and sham control animals expressed as **(C)** mean titers ± SD and in **(D)** longitudinal plots of individual animals. The red arrows and vertical fine dashed lines indicate the start of dexamethasone treatment.

In this study, we examined the immunogenicity and protective efficacy of an NXT-2a based vaccination strategy in the context of drug-induced immunosuppression. The affinity tag-free version of NXT-2, NXT-2a is approximately ~10 kDa and is recognized by sera from an NHP immunized with NXT-2 (**Figure 1A**). To confirm the immunogenicity of this modified antigen, healthy animals were vaccinated with NXT-2a (100µg) + Alhydrogel 2% (0.5mg Al^3+^) at 8 and 2 weeks prior to immunosuppression, 6 weeks apart (**Figure 1B**, NXT-2a Immunized). Three sham control animals were vaccinated with PBS + Alhydrogel 2% (0.5mg Al^3+^) at similar intervals and a fourth sham control animal 39635 received no vaccine. In NXT-2a immunized animals mean plasma NXT-2a IgG titers (± SD) peaked at four weeks (31,437 ± 59,060) following initial immunization and achieved robust titers (1,664,000 ± 443,405) two weeks after boosting (**Figures 1C, D**), demonstrating comparable immunogenicity to NXT-2.

To induce immunosuppression and render animals susceptible to *Pneumocystis* infection, vaccinated animals were then treated with dexamethasone (1.3-1.6mg/kg/day) beginning two weeks after the first boost. Sham control, 39635, initiated dexamethasone treatment ~4 weeks prior to the remainder of the cohort to function as a “seeder” to hasten infection within the co-housed experiment. Titers declined to 29,500 ± 34,307 within 4 weeks of dexamethasone treatment (**Figures 1C, D**). To determine if anti-*Pneumocystis* humoral immunity can be boosted in the context of drug-induced immunosuppression, NXT-2a immunized macaques were therapeutically immunized at 4 and 8 weeks following the start of dexamethasone treatment with NXT-2a (100µg) + Alhydrogel 2% (0.5mg Al^3+^) whereas sham controls received PBS + Alhydrogel 2% (0.5mg Al^3+^) or no vaccine (39635 only). After a third vaccination at 4 weeks of immunosuppression, 2 of 4 macaques responded to therapeutic boosting with NXT-2a (**Figure 1D**). Titers in NHP 40056 (**Figure 1D**, solid orange box) increased 5.9-fold between 4 and 6 weeks from 4,000 to 24,000 RET. Titers in NHP 40053 (**Figure 1D**, solid green box) showed a 1.2-fold increase during the same period from 80,000 to 96,000 RET. After a fourth vaccination at 8 weeks of immunosuppression, 4 of 4 animals responded to therapeutic boosting. Therapeutic vaccination increased mean titers approximately 6.9-fold between 8 and 10 weeks of immunosuppression from 37,250 ± 51,090 to 256,000 ± 78,383 RET. Mean NXT-2a IgG antibody titers remained above 10^4^ RET until the end of the study, 12 weeks after the start of immunosuppression (58,666 ± 40,266). Throughout our immunogenicity studies we did not observe any significant swelling, redness, or adverse reactions associated with NXT-2a immunization. These data demonstrate that NXT-2a is safe, highly immunogenic in healthy macaques, and a repeated boosting strategy maintains mean titers above 10^4^ RET for 12 weeks during dexamethasone-induced immunosuppression.

### 3.2 Effects of dexamethasone treatment on lymphocyte kinetics in NXT-2a and sham immunized macaques

**Figure 2:**
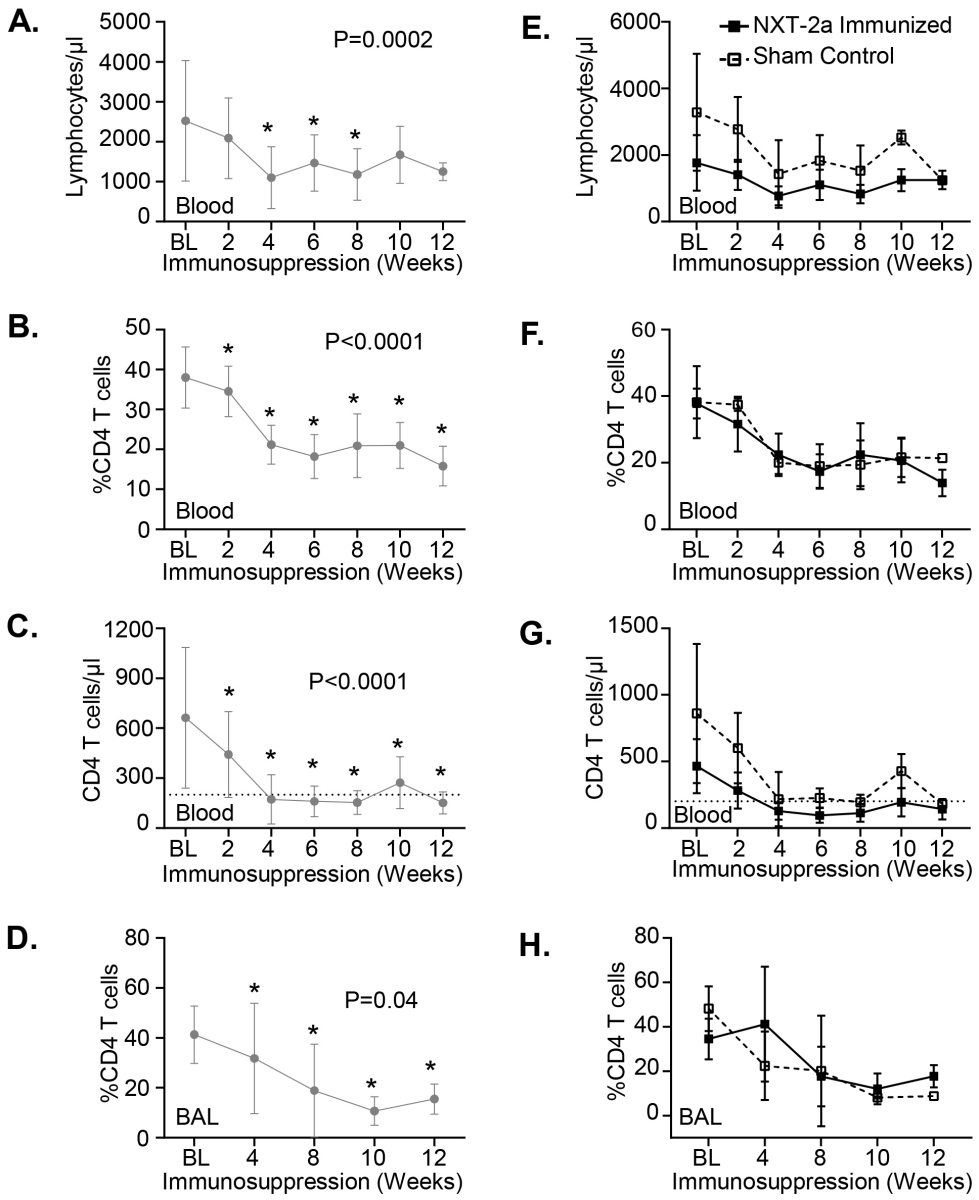
Lymphocyte and CD4 T cell populations in the blood and bronchoalveolar lavage (BAL) throughout dexamethasone treatment. Kinetics of lymphocyte depletion in **(A-D)** all dexamethasone treated macaques (grey circles) and in **(E-H)** NXT-2a immunized (closed box) *vs* Sham control (open box) animals. **(A, E)** Lymphocyte count, **(B, F)** CD4 T cell frequency and **(C, G)** cell number in the peripheral blood. **(D, H)** CD4 T cell frequency in the bronchoalveolar lavage (BAL). **(A-D)** Whole population kinetics were analyzed using repeated measures mixed modeling analysis (P-values are indicated) and Dunnett’s test for multiple comparisons. *P<0.05, indicates values that differ significantly from baseline (BL) in Dunnett’s *post-hoc* analyses. **(E-H)** Multiple Mann-Whitney tests were performed to evaluate the differences between NXT-2a immunized and Sham controls. Data represents the mean ± SD.

To confirm the immunosuppressive effects of dexamethasone throughout the course of this study, we monitored changes in immune subsets in the peripheral blood and BAL in all animals (**Figures 2A–D**). We observed significant longitudinal declines in lymphocytes (**Figure 2A**, P = 0.0002) and in the frequency (**Figure 2B**, P<0.0001) and cell number (**Figure 2C**, P<0.0001) of CD4 T cells in the peripheral blood. We also observed a decline in the frequency of CD4 T cells at the site of *Pneumocystis* infection in BAL samples (**Figure 2D**, P = 0.04). These data are consistent with previous observations reported in Japanese and Rhesus macaques similarly treated with dexamethasone (26). We did not observe significant differences in lymphocyte numbers, CD4 frequency, and CD4 T cell numbers between NXT-2a immunized or sham control animals (**Figures 2E–H**), indicating that both cohorts were similarly immunosuppressed throughout dexamethasone treatment. Longitudinal plots for individual animals are displayed in **Supplementary Figure 1**.

### 3.3 NXT-2a immunization is protective against *Pneumocystis* infection during drug induced immunosuppression

**Figure 3:**
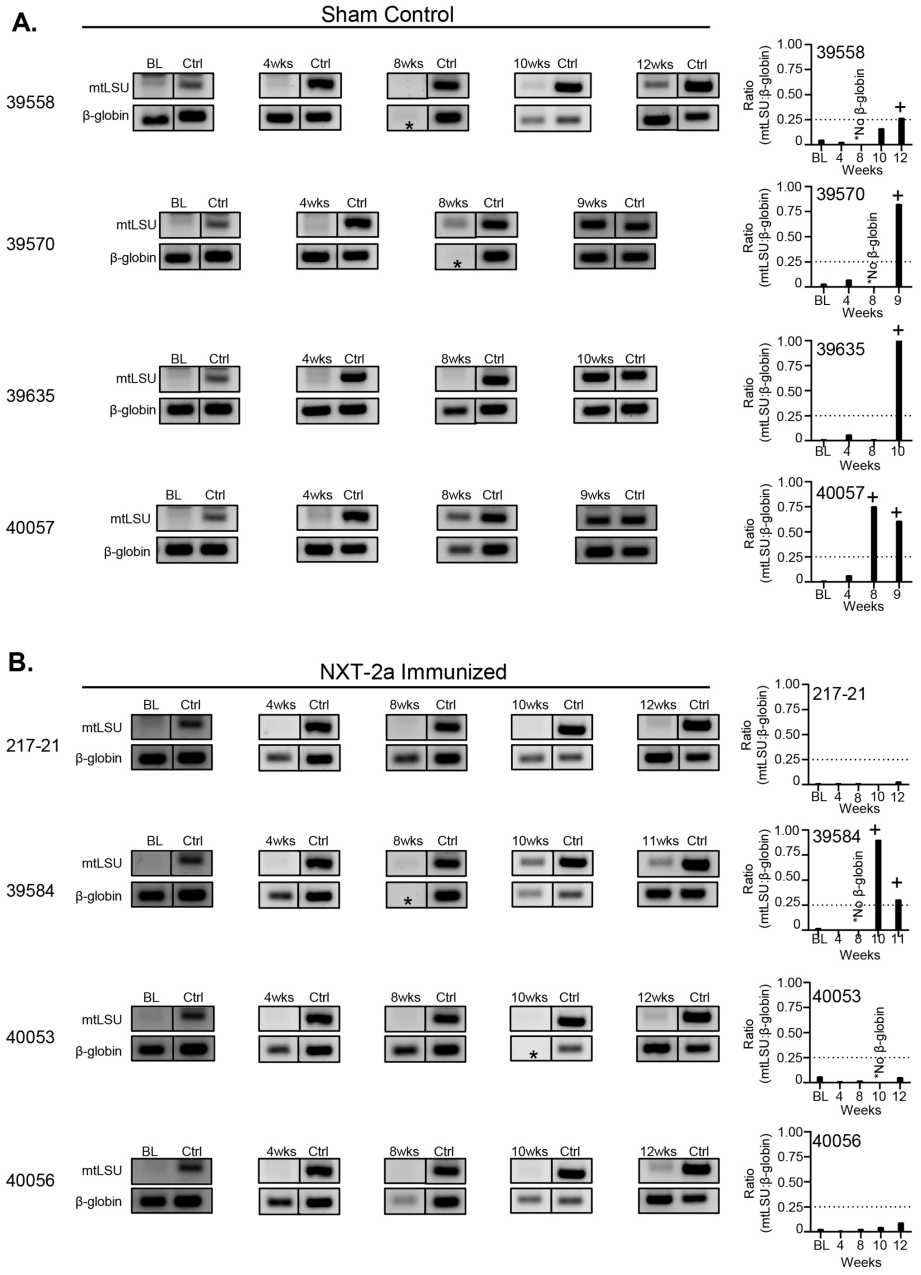
*Pneumocystis*-specific PCR of bronchoalveolar lavage samples throughout dexamethasone induced immunosuppression in NXT-2a immunized and sham control macaques. Gel analysis of mtLSU and β-globin PCR reaction products of **(A)** Sham control and **(B)** NXT-2a Immunized cohorts. Asterisks (*) indicate gel products in which β-globin was not detected. Positive control (Ctrl) reactions were performed in tandem at the indicated timepoints. Bar graphs represent densitometry analysis of PCR gel products expressed as a ratio of mtLSU:β-globin. Dashed lines on graphs indicate the threshold of *Pneumocystis* infection (+) at 0.25.

**Figure 4:**
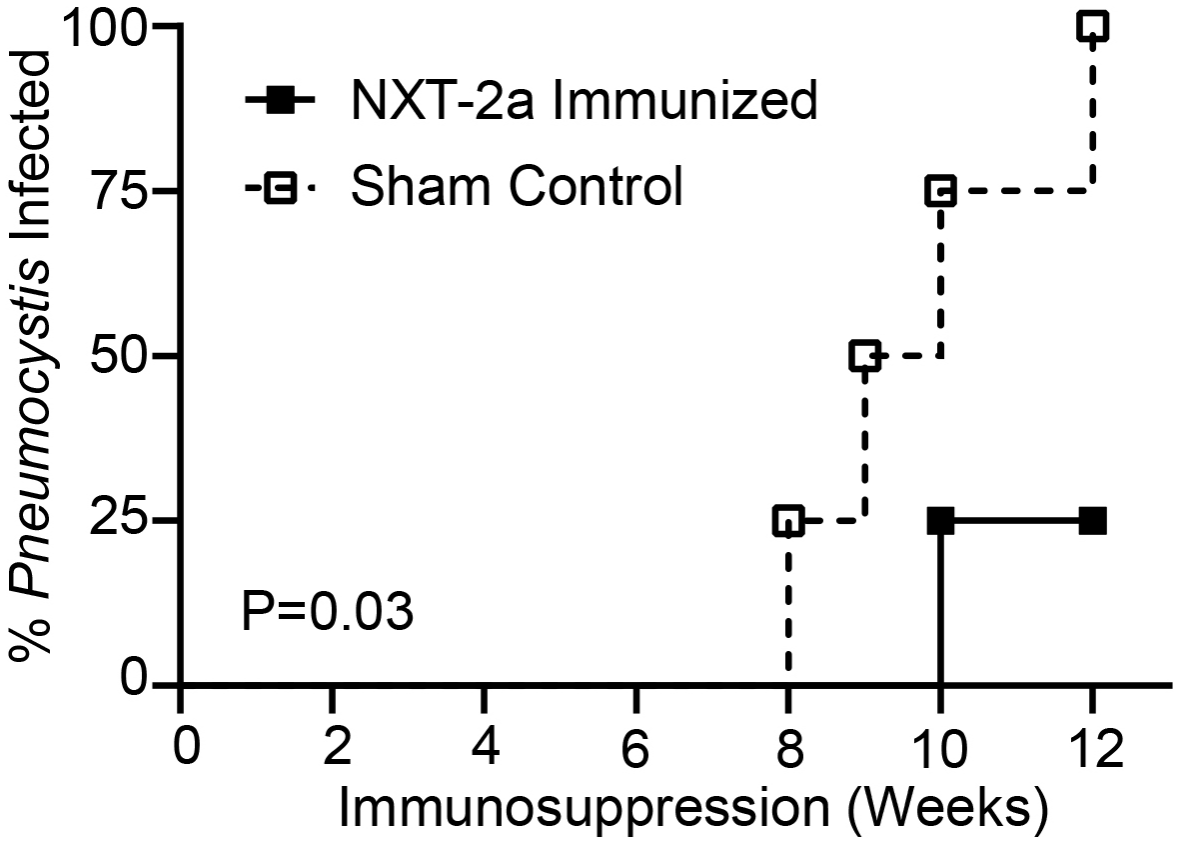
Incidence of *Pneumocystis* infection throughout dexamethasone induced immunosuppression in NXT-2a immunized and Sham control Japanese macaques. A diagnosis of *Pneumocystis* infection was made through the detection of the mtLSU gene in PCR amplified samples and subsequent densitometry analysis in samples with a ratio of mtLSU:β-globin greater than 0.25. Significance was determined by Log-rank test.

We have previously established that *Pneumocystis* infection and colonization can be reliably diagnosed in dexamethasone and SIV immunosuppressed macaques through the detection of *Pneumocystis* DNA in PCR amplified BAL samples (22, 24, 26). To monitor *Pneumocystis* infection, throughout the course of this study, longitudinal BAL sampling was performed at baseline and throughout immunosuppression. At the start of the study, we confirmed that all animals were uninfected with *Pneumocystis*. Following the start of dexamethasone treatment, we observed *Pneumocystis* infection in sham control macaques 39558, 39570, 39635, and 4007 at 12, 9, 10, and 8 weeks of immunosuppression, respectively (**Figure 3A**; **Table 1**) and in NXT-2a immunized macaque 39584 at 10 weeks (**Figure 3B** second row; **Table 1**). At study termination, only 25% (1 of 4) NXT-2a immunized animals became *Pneumocystis* infected compared to 100% (4 of 4) of sham controls (**Figure 4**, P = 0.03, **Table 1**), despite being comparably immunosuppressed (**Figure 2**).

**Table 1:** Summary of *Pneumocystis* BAL PCR results.

Macaque	Gender	Age (Years)	*Pneumocystis* status* at experiment end	Timepoint *Pneumocystis* infection, weeks of IS
Sham Control				
39558	Female	4.6	Infected	12
39570	Female	4.6	Infected	9
39635†	Male	4.4	Infected	10
40057	Male	4.4	Infected	8
NXT-2a Immunized				
217-21	Female	8.4	Colonized	--
39584	Male	4.6	Infected	10
40053	Female	4.4	Colonized	--
40056	Female	4.4	Colonized	--

*Pneumocystis status was determined by PCR analysis of BAL samples.

† “Seeder” animal.

IS, immunosuppression.

**Figure 5:**
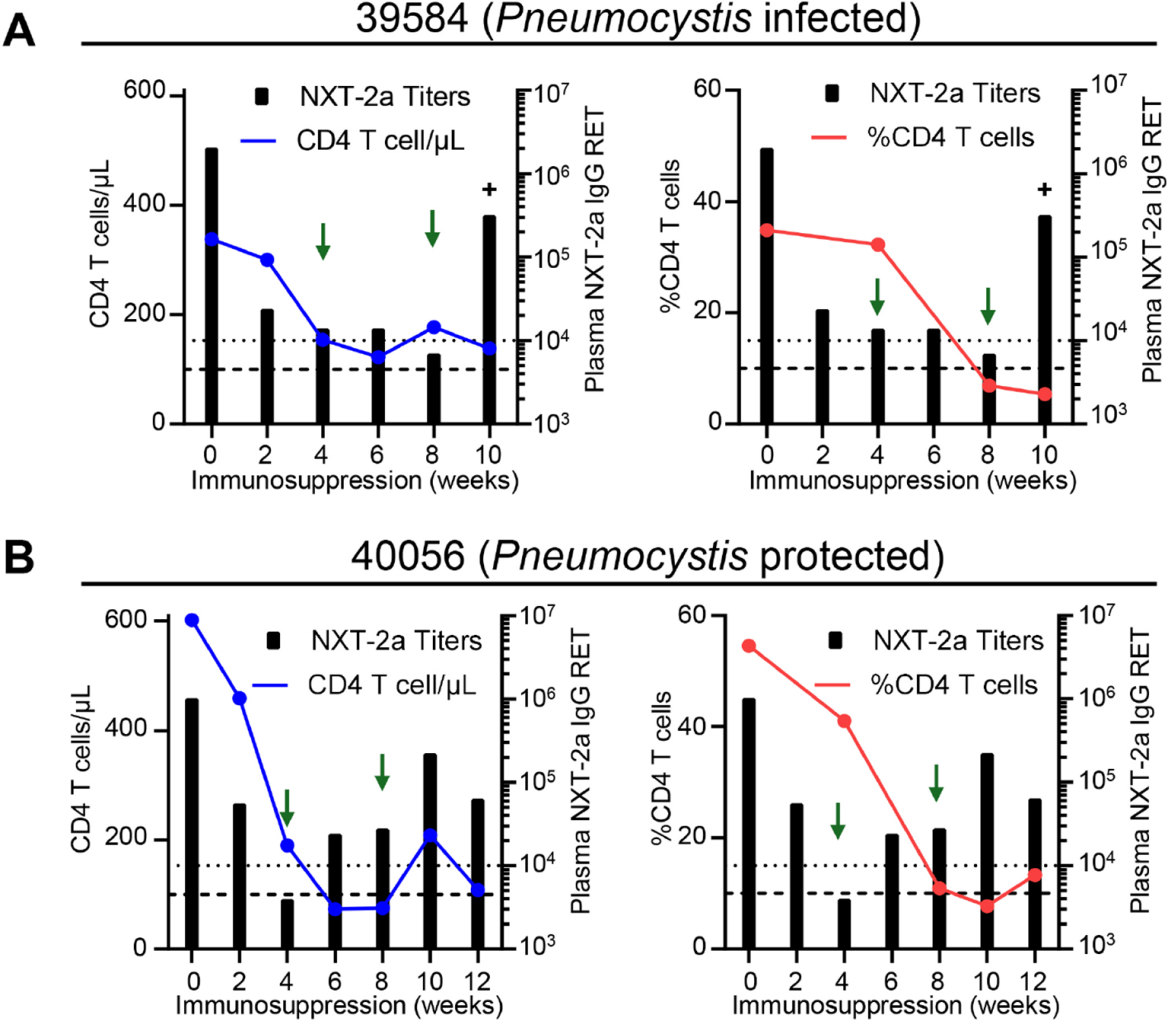
NXT-2a antibody responses in animals with severe CD4 T cell depletion. CD4 T cell depletion profiles and NXT-2a antibody responses throughout dexamethasone treatment and therapeutic boosting. **(A)** *Pneumocystis* infected NHP 39584. **(B)** *Pneumocystis* protected NHP 40056. (Left panels) Number of CD4 T cells in the peripheral blood and plasma NXT-2a IgG RET throughout immunosuppression. (Right panels) Frequency of CD4 T cells in the bronchoalveolar lavage and plasma NXT-2a IgG RET throughout immunosuppression. Thick dashed lines indicate 100 CD4 T cells/µl or 10% CD4+ T cells. Dotted dashed lines indicate 10^4^ RET. Green arrows indicate therapeutic boosting at 4 and 8 weeks of immunosuppression. Plus sign (+) indicates the timepoint of *Pneumocystis* infection in NHP 39584.

We have previously reported that low antibody titers against below 10^4^ IgG RET against PC.KEX1 are predictive of *Pneumocystis* susceptibility following SIV immunosuppression (21). In the single NXT-2a immunized macaque that became *Pneumocystis* infected, NHP 39584, plasma NXT-2a IgG antibody titers dipped below 10^4^ RET after 8 weeks of dexamethasone treatment, approximately 2 weeks prior to infection. At this same timepoint, 39584 also experienced a decline in CD4 T cell numbers to <200 cells/µl (**Figure 5A** left panel) and the CD4 frequency in the BAL was < 10% (**Figure 5A** right panel), reflective of a severe state of immunosuppression. In contrast, NXT-2a immunized NHP 40056, maintained plasma NXT-2a IgG titers above 10^4^ RET when similarly immunosuppressed between 8–12 weeks of dexamethasone treatment (**Figure 5B**) and remained uninfected. These data indicate that our vaccination strategy with therapeutic boosting during immunosuppression is antibody mediated and protective against *Pneumocystis* infection.

## 4 Discussion

The expanding use of corticosteroids and immunomodulatory drugs in solid-organ and hematopoietic transplant recipients, cancer patients, and persons with inflammatory autoimmune disorders likely contributes to the increase the number of persons at risk of *Pneumocystis* associated pulmonary disease (8, 9, 29). *Pneumocystis* pneumonia risk is especially problematic in individuals receiving high dose corticosteroids for greater than 4 weeks, have CD4 T cell counts of <200cells/µl, and are TMP-SMX prophylaxis non-adherent (9, 30–32). Using a highly relevant pre-clinical model with natural transmission by airborne exposure (26), we sought to evaluate the immunogenicity and protective efficacy of a pan-fungal vaccination strategy in a NHP model of *Pneumocystis* infection in the context of drug-induced immunosuppression.

In the current study, healthy macaques were immunized with an affinity tag-free variant of NXT-2 (18), NXT-2a, 8 and 2 weeks prior to immunosuppression. Immunization with NXT-2a during immunocompetency induced robust NXT-2a IgG titers above 10^6^ RET. These results were consistent with prior studies with NXT-2 in healthy rhesus macaques (18) and confirmed the immunogenicity of our modified antigen. To test the hypothesis that NXT-2a immunization could boost titers in the context of drug-induced immunosuppression, we administered a third and fourth vaccine at 4 and 8 weeks of dexamethasone treatment, respectively. During immunosuppression, NXT-2a antibody titers were boosted in 2 of 4 NXT-2 immunized macaques after a 3^rd^ immunization and 4 of 4 after 4^th^ immunization. Mean NXT-2a IgG antibody titers remained above 10^4^ RET until the end of the study. NXT-2a immunization afforded significant protection against *Pneumocystis* infection when compared with sham controls (1 of 4 (25%) NXT-2a immunized *vs*. 4 of 4 (100%) Sham controls). This vaccine strategy was even protective in a profoundly immunosuppressed animal with only 100–200 cells/µl (NHP 40056).

We have previously reported that antibody titers above 10^4^ IgG RET against a similar antigen PC.KEX1, in healthy macaques correlate with protection against *Pneumocystis* co-infection following SIV-induced immunosuppression (21). In the single NXT-2a immunized macaque that became infected (NHP 39584) plasma NXT-2a antibody levels subsequently declined below 10^4^ RET approximately two weeks prior to a positive diagnosis. This animal eventually responded to a second therapeutic immunization, but due to the gap in coverage this delayed response was not sufficient to prevent infection. Interestingly, when we re-evaluated this animal by PCR a week after diagnosis and boosting, the ratio of mtLSU:β-globin declined from 0.90 to 0.30. We speculate that the rise in NXT-2a antibody levels induced by therapeutic boosting may have prolonged control of *Pneumocystis* infection. These results demonstrate effective boosting and protection by our NXT-2a based vaccine strategy in drug-immunosuppressed macaques.

Immunosuppressive therapies such as dexamethasone and other glucocorticoids induce acute dysfunction in both innate and adaptive immune compartments that may hinder the performance of traditional vaccine strategies in these populations even more so than in persons with CD4 targeted depletion due to HIV infection. Due to the assumed strain on vaccine durability in immunosuppressed individuals in general, current guidelines for *Pneumocystis* susceptible non-HIV populations, such as solid organ transplant recipients, recommend patients be up to date on vaccinations prior to transplantation and receive booster doses between 3–12 months post-transplant, except in the case of viral or live attenuated vaccines and the influenza vaccine (33). A multi-center cohort study found that administration of inactivated influenza vaccine within the first three months of transplantation is safe and effective (34). Due to seasonal exposure, inactivated influenza vaccines are now routinely recommended as early as one-month post-transplant with consideration of repeating additional doses at two and three months if disease transmission continues (33, 35).

In contrast to the seasonality of influenza exposure, *Pneumocystis* is ubiquitous, and disease has been demonstrated to arise through exposure to other *Pneumocystis* carrying hosts (5, 36). *Pneumocystis* pneumonia clusters have been frequently found within transplant centers due to nosocomial exposure (37, 38). Given the safety of the influenza and many other inactivated vaccines administered post-transplant, the ubiquitous nature of *Pneumocystis*, and our observations that NXT-2a immunization limits *Pneumocystis* infection, we argue that the vaccine strategy used in this study is safe and highly relevant. The data presented herein accurately mimics the challenges to immunization faced by immunosuppressed clinical populations. We hypothesize that NXT-2a could be administered to patients before and after the start of immunosuppression, as well as to close contacts of patients, to limit *Pneumocystis* infection and exposure. Responses to therapeutic immunization administered after induction of immunosuppression therapy would also likely increase in amplitude and durability as regimens are tapered to maintenance levels. Furthermore, as a ‘pan-fungal’ vaccine candidate, this NXT-2a based immunization strategy may provide additional coverage against other invasive fungal pathogens such as *Aspergillus* spp., *Candida* spp., and more with demonstrated anti-NXT-2 antibody cross-reactivity (18).

There are limitations to our study. We did not evaluate the protective efficacy of the primary vaccine series (first and second vaccine) alone in the absence of therapeutic boosting during immunosuppression. In addition, we did not test the ability of our vaccine to generate a *de novo* memory response during immunosuppression. Studies to improve memory responses against NXT-2a and antibody titer durability in both immunocompetent and immunosuppressed conditions in larger cohorts are ongoing. Further studies investigating *de novo* memory response against NXT-2a in the context of drug-induced immunosuppression will help to establish the potential and limitations of NXT-2a based vaccination strategies in patients who are already severely immunosuppressed.

In summary, we report a novel vaccination strategy for the prevention of *Pneumocystis* infection in a NHP model of drug-induced immunosuppression. Our data demonstrates the importance of anti-NXT-2a antibodies in the control of *Pneumocystis* infection in the context of drug-induced immunosuppression. These data warrant future investigation of passive transfer studies with anti-NXT-2a antibodies in populations that are similarly immunosuppressed as well as in populations who are less likely to benefit from active immunization. These data may be of interest to investigators and clinicians interested in immunization strategies for vaccine preventable diseases in immunocompromised populations.

## Data Availability

The raw data supporting the conclusions of this article will be made available by the authors, without undue reservation.
