# Correction: Protective efficacy of a ‘pan-fungal’ vaccination strategy against experimental *Pneumocystis* infection in drug-immunosuppressed macaques

**DOI:** 10.3389/fimmu.2026.1798599

**Published:** 2026-02-17

**Authors:** Whitney Rabacal, Anna Hu, Gabrielle Kirton, Taylor I. Chapman, Daniel Wychrij, Kwadwo O. Oworae, Karen A. Norris

**Affiliations:** Center for Vaccines and Immunology, Department of Infectious Diseases, University of Georgia, Athens, GA, United States

**Keywords:** dexamethasone, immunosuppression, macaque, non-human primate, NXT-2, pan-fungal vaccine, pneumocystis

There was a mistake in **Figure 5** as published. The figure keys were incorrectly labeled as “KEX1 Titers” (adjacent to the black bars) instead of “NXT-2a Titers” in all four panels (**Figures 5A, B**, left and right panels). The corrected **Figure 5** appears below.

**Figure 5:**
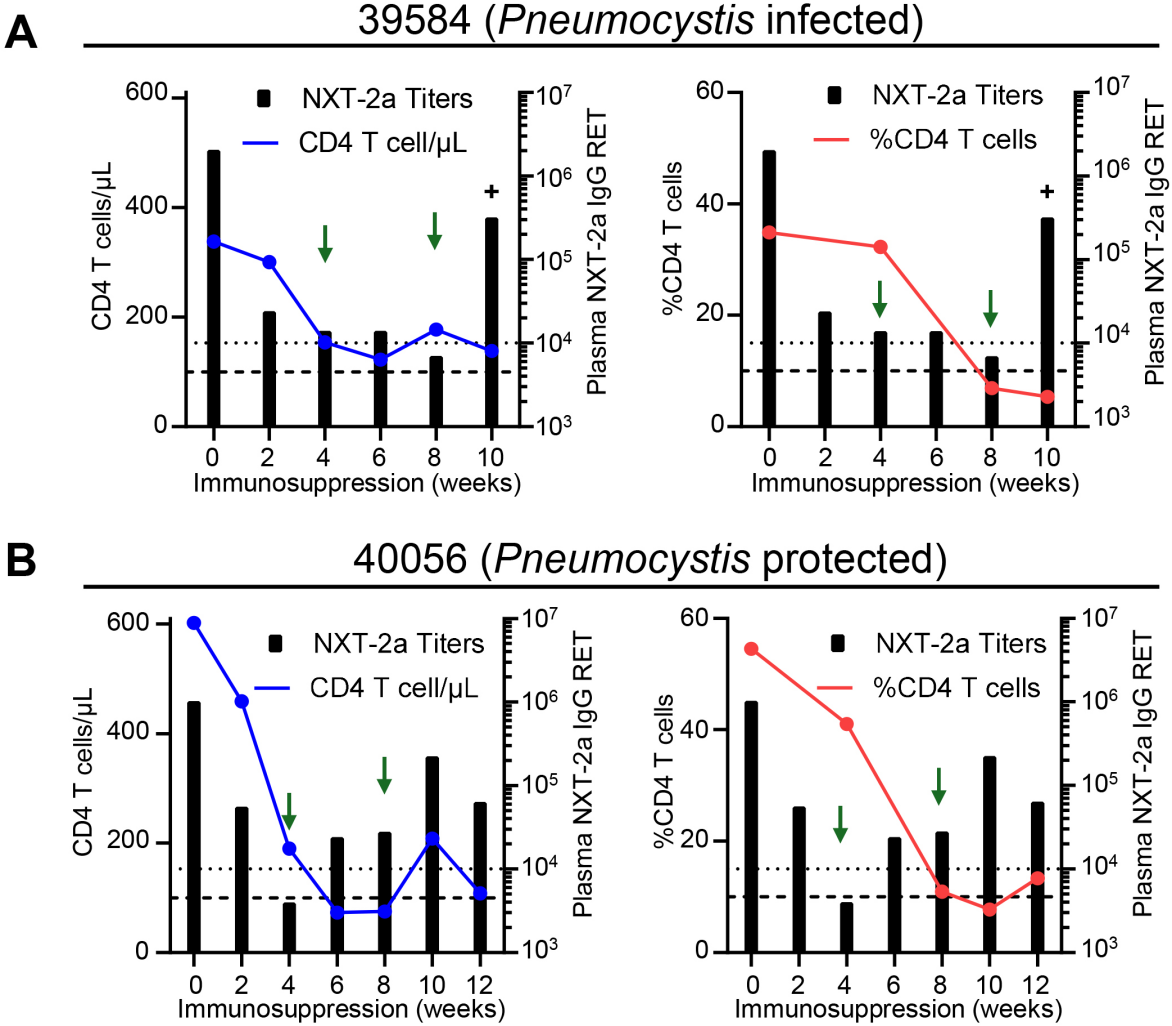
NXT-2a antibody responses in animals with severe CD4 T cell depletion. CD4 T cell depletion profiles and NXT-2a antibody responses throughout dexamethasone treatment and therapeutic boosting. **(A)** *Pneumocystis* infected NHP 39584. **(B)** *Pneumocystis* protected NHP 40056. (Left panels) Number of CD4 T cells in the peripheral blood and plasma NXT-2a IgG RET throughout immunosuppression. (Right panels) Frequency of CD4 T cells in the bronchoalveolar lavage and plasma NXT-2a IgG RET throughout immunosuppression. Thick dashed lines indicate 100 CD4 T cells/µl or 10% CD4+ T cells. Dotted dashed lines indicate 10^4^ RET. Green arrows indicate therapeutic boosting at 4 and 8 weeks of immunosuppression. Plus sign (+) indicates the timepoint of *Pneumocystis* infection in NHP 39584.

The original version of this article has been updated.

