# Supplementary figures and images for: Protective efficacy of a ‘pan-fungal’ vaccination strategy against experimental Pneumocystis infection in drug-immunosuppressed macaques
Source: Front Immunol. 2026 Jan 16;16:1729080. doi: 10.3389/fimmu.2025.1729080 (PMC12855516; doi:10.3389/fimmu.2025.1729080)

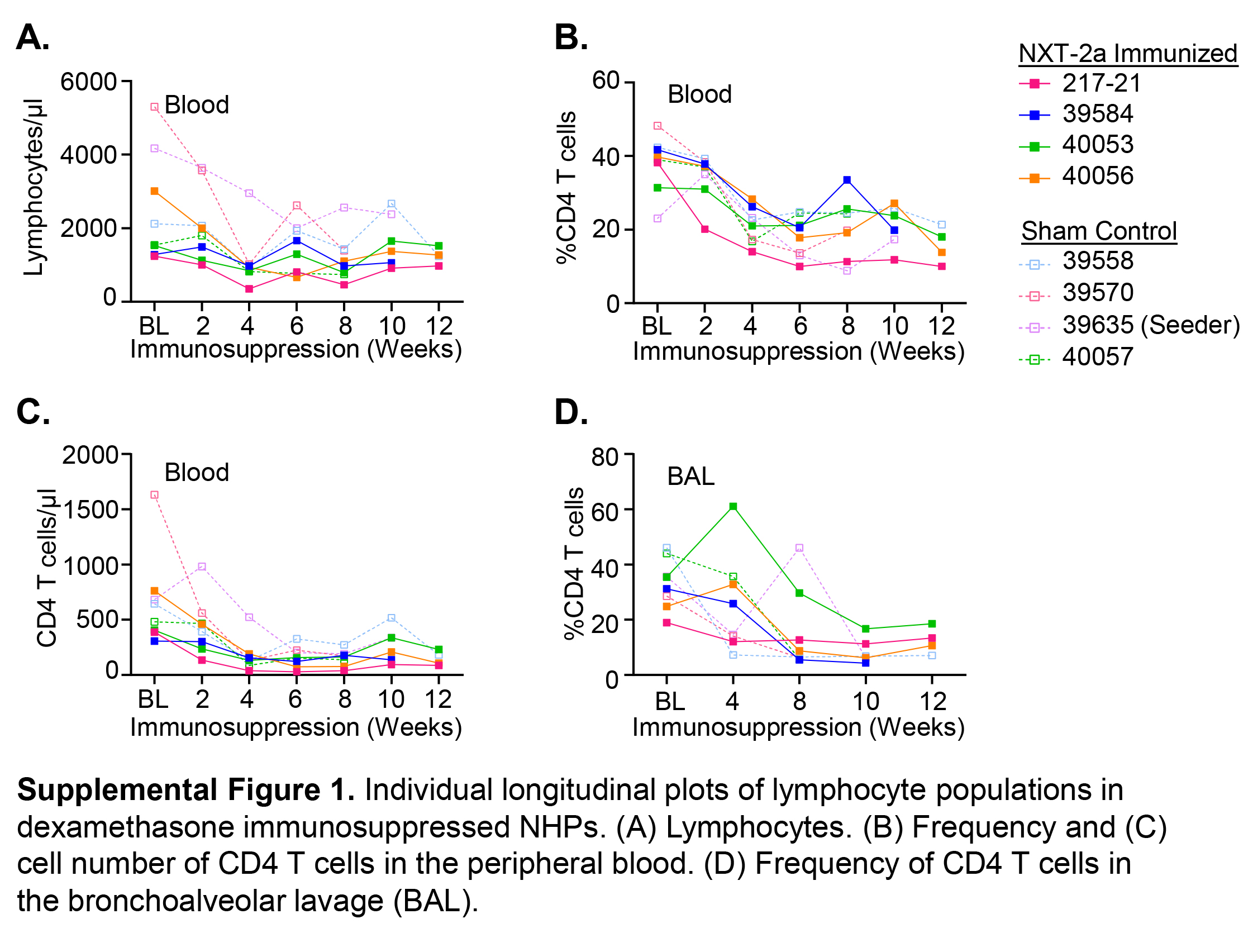

Supplement: Supplementary file 1 [file Image1.jpeg]
